# Bioremediation and tolerance of zinc ions using *Fusarium solani*

**DOI:** 10.1016/j.heliyon.2020.e05048

**Published:** 2020-09-28

**Authors:** Manal T. El Sayed, Ashraf S.A. El-Sayed

**Affiliations:** Botany and Microbiology Department, Faculty of Science, Zagazig University, 44519, Egypt

**Keywords:** Biochemistry, Biotechnology, Materials science, Microbiology, Metabolite, Pharmaceutical science, Biomedical engineering, *Fusarium solani*, Zn(II), Stress, Antioxidant enzymes, Biosorption

## Abstract

Evaluating the mechanism of tolerance and biotransformation Zn(II) ions by *Fusarium solani* based on the different physiological was the objective of this work. The physical properties of synthesized ZnONPs was determined by UV-spectroscopy, transmission electron microscope, and X-ray powder diffraction. The structural and anatomical changes of *F. solani* in response to Zn(II) was examined by TEM and SEM. From the HPLC profile, oxalic acid by *F. solani* was strongly increased by about 10.5 folds in response to 200 mg/l Zn(II) comparing to control cultures. The highest biosorption potential were reported at pH 4.0 (alkali-treated biomass) and 5.0 (native biomass), at 600 mg/l Zn(II) concentration, incubation temperature 30 °C, and contact time 40 min (alkali-treated biomass) and 6 h (native biomass). From the FT-IR spectroscopy, the main functional groups implemented on this remediation were C–S stretching, C=O C=N, C–H bending, C–N stretching and N–H bending. From the EDX spectra, fungal cellular sulfur and phosphorus compounds were the mainly compartments involved on ZN(II) binding.

## 1 Introduction

Anthropogenic and natural activities discharge poisonous heavy metals into the surroundings (Singh et al., 2018), that might be non-degradable and long persisting in the environment (Taamalli et al., 2014), causing sever harmful influences on the environment, and food chains (Mahmood and Malik, 2014). Zinc (Zn(II)) is an important microelement for livings, however, the elevated levels of Zn (II) are harmful and threaten the lives of many organisms (Salem and Fouda, 2020). Normal Zn(II) levels in soil and fresh water are usually ranged 10–300 mg/kg and <0.1–50 μg/l, respectively. Due to the anthropogenic pollution and natural processes, total Zn(II) concentrations in soil and freshwater are raised up to 35000 mg/kg and 3900 μg/l, respectively (WHO, 2001). The principal sources of Zn(II) are steel and iron production, mining, zinc-containing pesticides, fertilizers, and corrosion of galvanized structures (Ferreira et al., 2018). The symptoms of Zn(II) poisoning are gastrointestinal pain, diarrhea, and vomiting, due to the usage of water kept in galvanized units. Thus, Zn(II) toxicity could be a field of concern for the environmental, organic process and ecological reasons (Jaishankar et al., 2014).

Several ancient strategies like electrodialysis, ion exchange, membrane filtration, and coagulation-flocculation have been applied for the metal alleviation. Among the drawbacks of these approaches are incomplete elimination, polluted sludge formation, high cost, high energy conditions, and membranes plugging (Firdousi, 2017; Ayangbenro and Babalola, 2017; Aziz et al., 2015). Bioremediation is the most feasible and eco-friendly approach for metals removal via bioleaching, intracellular uptake, redox reactions, biomineralization, and biosorption (Hamba and Tamiru, 2016). Mycoremediation is the most effective strategy for removal of heavy metals, with special interest for production of various enzymes, organic acids, and economical metabolites (Bosco and Mollea, 2019). Moreover, fungi displayed a superior metal-binding features and resistance to metals and unfavorable conditions. Fungal cell wall is the primary interaction site with the metal, composed of chitin, chitosan, glucan, polysaccharides, lipids and proteins with enormous functional groups (hydroxyl, carboxylate, sulfate, phosphate, and amino) (Abbas et al., 2014). Several fungal species were reported to be involved in detoxification of various metals contaminants, for example, removal of V(II) by *Aspergillus terreus*, *Cladosporium cladosporioides*, *Paecilomyces lilacinus*, *Penicillium citrinum* and *Rhizopus arrhizus* (Ceci et al., 2012), Cu(II) by *Trichoderma viride* (Wang and Wang, 2013), Ni(II) by *T. harzianum* (Cecchi et al., 2017a), Ag(I) by *A. alliaceus*, *T. harzianum*, and *Clonostachys rosea* (Cecchi et al., 2017b), Pb(II) by different *Pleurotus* sp (Dulay et al., 2015), Zn(II) by *P. janthinellum*, *P. olsonii* and *P. waksmanii* (Di Piazza et al., 2018), Cd(II) by *A. versicolor* (Fazli et al., 2015), Fe(III), Mn(II), Cu(II), Zn(II), and Pb(II) by *Mucor circinelloides* (Zhang et al., 2017) were studied.

Microbes display various approaches to overcome the hazardous effects of metals. The metal resistance mechanism are mainly avoidance and tolerance (Fouda et al., 2018; Nedkovska and Atanassov, 1998). Fungal tolerance mechanisms involve mobilization, immobilization, and biotransformation. Mobilization of metals occurs by heterotrophic and auxotrophic leaching, complexation/chelation by various metabolites, whereas immobilization occurs due to metal sorption with the biomass or exopolymers, intracellular sequestration and precipitation as organic and inorganic compounds (Singh et al., 2018). Some microorganisms show an excellent bio-transformational efficiency, transforming the poisonous chemical to nontoxic forms that permit the microbe to overcome the toxicity of pollutants (Mishra and Jha, 2009; El-Sayed, 2011; El-Sayed and Shindia, 2011; Mohamed et al., 2019). They released energy from the reduction/oxidation of As(V)/As(III) are mainly implemented on their growth (Oremland and Stolz, 2003). The potency of As(V) reduction were reported for *T. asperellum*, *P. janthinellum*, and *Fusarium oxysporum* (Su et al., 2012). This study aimed to assess *F. solani* for its tolerance towards Zn(II). The biosorption of Zn(II) by living and alkali-treated *F. solani* under various factors studied. The mechanism of tolerance and biosorption elucidated by FTIR, EDX, SEM, TEM, and XRD.

## 2 Materials and methods

### 2.1 Fungal isolate and zinc tolerance assay

*Fusarium solani* KJ 623702 had been isolated and molecularly identified from our previous work (El-Sayed, 2014, El-Sayed and El-Sayed, 2020a, b). The strain conserved on PDA slants at 4 °C, supplemented with ZnSO_4_.2H_2_O to give final desired concentrations (0, 2000, 4000, 6000, 8000, 9000, 10000, 11000, and 12000 mg/l), then poured to the petri plates. The plates were centrally inoculated with the fungal inoculum plug (6 mm of 7 days old cultures), incubated at 25 °C for 6 days, and the fungal growth was measured. The Minimum Inhibitory Concentrations (MIC) was denoted by the lowest Zn (II) concentration that inhibited the growth of *F. solani* (Shaheen et al., 2019; Sabatini et al., 2016, El-Sayed et al., 2012a, b). All the experiments throughout the recent study were performed in triplicates.

### 2.2 Scanning electron microscopy (SEM) analysis

For evaluation of the morphological deformation in response to Zn(II)-stress, the fungus was treated at the sub-MIC dose, incubated, and investigated with SEM. The mycelia were fixed in 2.5% glutaraldehyde for 24 h at 4 °C, post-fixed in osmium tetraoxide (1.0%) for 1 h, and then dehydrated with acetone (El-Sayed et al., 2014). The gold coated samples were examined by the scanning electron microscope (JEM-1200XII).

### 2.3 Energy dispersive X-ray (EDX) microanalysis

The collected mycelia were treated with Zn(II) at the sub-MIC, incubated, and subjected to EDX micro-analysis for quantitative elemental analysis by X-ray microanalyzer (model Oxford 6587 INCA X-sight) connected to scanning electron microscope.

### 2.4 Transmission electron microscopy (TEM) analysis

For estimating the cytomorphological changes caused by Zn(II), the fungus was incubated with Zn(II) at the sub-MIC dose, incubated, and the cellular organelles were investigated (El-Sayed and Ali, 2020). Samples were primary fixed with 2.5 % glutaraldehyde for 3 h at 4 °C, washed with 0.2 M phosphate buffer (pH 7.4) for 30 min, post-fixed in osmium tetraoxide (1.0 %) for 2 h at 4 °C, washing with phosphate buffer for 30 min. Samples were dehydrated in a gradient concentrations of ethanol (50–100%), transferred via a three changes of acetone: ethanol (1:2, 1:1, and 2:0) for 10 min, then embedded in epoxy medium. A diamond knife sectioned the blocks into ultrathin sections of 70 nm and placed on copper grids. The sections contrasted by uranyl acetate and by lead citrate for 30 min. Transmission and photographing was conducted by the electron microscope.

### 2.5 Growth response of *F. solani* in Zn(II)-enriched media

To explore the response of *F. solani* to Zn(II) stress, the tolerance index (TI), dry weight, percentage of removal, contents of H_2_O_2,_ lipid peroxidation, the concentrations of antioxidants, soluble protein, and thiol, and polyphenol oxidase (PPO) activity were determined (El-Sayed et al., 2015a,b,c,d,e). *F. solani* evaluated for Zn(II) tolerance index (TI) at concentrations extending from 1000 to 9000 mg of Zn(II)/l. Zn(II)-free medium was considered as a control. PDA plates were inoculated at the middle with six mm agar plugs and kept at 25 °C for ten days. TI was determined from the radial growth of Zn(II)-stressed strain divided by the growth in the Zn(II)-free plates. The TI was evaluated as follows: 0.00 to 0.39 (very low tolerance), 0.40 to 0.59 (low tolerance), 0.60 to 0.79 (moderate tolerance), 0.80 to 0.99 (high tolerance) and 1.00 to >1.00 (very high tolerance) (Oladipo et al., 2018).

To investigate Zn(II) bioremoval and the impact of Zn(II) on the fungal dry weight, sterilized ZnSO_4_.2H_2_O solutions were aseptically supplemented to the sterile PD broth (pH was maintained at 5.8 by the standard solution of 0.1 N NaOH/HCl) to get the final concentration of 0, 200, 500, 1000, 2000, 4000, 6000, 7000, and 8000 mg/l, then inoculated with spore suspension (10^6^/ml), and incubated for seven days at 25 °C and 140 rpm. During the growth, the white coalescence was notice, suggesting the reduction of Zn(II) and the formation of zinc oxide nanoparticles (ZnONPs). The biomass was separated by centrifugation and dried at 60 °C, the filtrates were utilized for characterization of ZnONPs by UV-Visible spectroscopy, TEM analysis (JEOL TEM-1400), and X-ray powder diffraction (XRD) (Broker D8 Advanced target Cu Koα powder diffractometer (λ = 1.5418 °A) (El-Sayed and El-Sayed, 2020a, b). For the TEM analysis, the samples were loaded on carbon-coated grids and dried, a thin film on glass slides was dried at 45 °C and used for XRD. The residual Zn(II) was measured with atomic absorption spectrophotometer (Unicam 969) (El-Sayed and El-Sayed, 2020a, b). The efficiency of removal (E) determined according to the following equation:

E = [(Ci − Cf)/Co] ×100

Where Ci and Cf are initial and residual concentrations of Zn(II) (mg/l), respectively.

### 2.6 Antioxidants and enzymatic activities

For antioxidative studies, the fungal biomass was pulverized in 50 mM phosphate buffer (pH 7.0) of 50 mM EDTA in an ice-cold mortar and centrifuged. The supernatants were used to clarify the tolerance mechanism.

#### 2.6.1 Polyphenol oxidase (PPO)

Samples (200 μl) subjected to the reaction with 5 U/ml horseradish peroxidase, guaiacol (0.2 mM), and catechol (10 mM) in one ml as a final volume, kept at 30 °C for 60 min and frozen for 10 min. The detected color conducted at 436 nm (Bergmeyer et al., 1974, El-Sayed et al., 2013a,b). The specific enzymatic activity expressed in enzyme units (the enzyme amount that released 1 μmol H_2_O_2_/min under optimum conditions)/mg protein/min.

#### 2.6.2 Catalase assay

the reaction solution containing 3 ml of 10 mM phosphate buffer pH 7, 0.2 ml of 0.2 M H_2_O_2_, and 0.1ml of the enzyme extract incubated for 10 min and absorbance measured at 240 nm (Abhishek et al., 2010; El-Sayed et al., 2016).

#### 2.6.3 Total antioxidant

The total antioxidant was estimated by ferric-thiocyanate method (Gupta et al., 2004, El-Sayed et al., 2017a,b). In brief, the supernatant (1 ml) was mixed with 0.2 ml ferrous chloride (20 mM) and 0.2 ml ammonium thiocyanate (30%) and kept for 10 min and red color measured at 500 nm.

#### 2.6.4 Assay of total thiol content

The total thiols were determined by Ellman's reagent (1959) with some modifications (El-Sayed et al., 2019a,b,c). The fungal extracts (3 ml) were mixed well with 2 ml phosphate buffer of pH 7.0 and 5.0 ml distilled water. Three milliliters of the mixture were shaken with 0.01 M DTNB (20 μl) and absorbance estimated at λ412 nm.

#### 2.6.5 Protein measurement

The soluble proteins was quantified by Folin's reagent (Lowry et al., 1951). Briefly, 1 ml of the prepared fungal extract was mixed with 1 ml of freshly prepared solution C (50:1 V/V, solution A to B), incubated for 15 min at room temperature. Folin's reagent (50 μl) was added to the mixture, shaking for 20 min, and the developed blue color was measured at A_650_ nm. The actual concentration of proteins was calculated using bovine serum albumin as authentic (El-Sayed et al., 2018a,b).

#### 2.6.6 Hydrogen peroxide (H_2_O_2_) content

The fungal mycelia were pulverized in 0.1% TCA, filtered, the mycelium extract (0.5 ml) was mixed with 2 ml of 1 M KI in bi-distilled water and 0.5 ml 100 mM potassium phosphate buffer (pH 6.8) and left for 1 h in dark (Alexieva et al., 2001). The hydrogen peroxide concentration was determined at 390 nm with baseline of TCA. Absorbance measured at 390 nm. From a standard curve prepared with known concentrations of H_2_O_2_, the amount of H_2_O_2_ expressed as μg/g of fresh weight.

### 2.7 Determination of malonyl dialdehyde (MDA) content (lipid peroxidation *product*)

In 5% TCA (1.5 ml), 0.2 gm of the mycelia were homogenized and centrifuged. A mixture composed of 0.5 ml of the supernatant, 1 ml of 20% TCA, and 0.5 % thiobarbituric acid (1 ml) put in water bath for 25 min at 95 °C, then cooled immediately and centrifuged. The absorbance was measured at 450, 532, and 600 nm (Li, 2000).

MDA(μm/ml) = 6.45 (A_532_- A_600_)-0.54 A_450_

To study the function of oxalic acid in Zn(II) tolerance, the concentrations of oxalic acid in Zn(II)-free and Zn(II)-stressed culture filtrates (4000 mg/l) were assessed by HPLC. The HPLC system consisted of a GBC UV/vis detector, GBC LC 1110 pump monitored by WinChrome chromatography (Kromasil column, 100 × 4.6 mm). The samples were eluted with 85% acetonitrile and 15% water at flow rate 1 ml/min. The concentration of oxalic acid was assessed at λ_254_ nm comparing to known concentrations of authentic oxalic acid.

### 2.8 Biosorption studies

#### 2.8.1 Preparation of the biosorbents

After culturing *F. solani* in PDB at 25 °C for 7 days under shaking conditions (120 rpm), the mycelia were harvested and rinsed with sterilized distilled water. Part of the mycelia was utilized for uptake analysis of the biosorption potency of biosorbents. The other part was treated by mixing the mycelia with NaOH (0.2N) for 1 h till neutral pH (Kapoor and Viraraghavan, 1998). All sorption measures were performed in 250 ml Erlenmeyer flasks of 50 ml of Zn(II) solutions at 140 rpm with pH range 2–6, biosorbent dose (1.0–5.0 g/l), metal concentration (200–700 mg/l), contact time (0–24h) and incubation temperature (10–60 °C). The working solutions were centrifuged to determine the residual Zn(II) concentration.

Biosorption capacity (q) = [(Ci − Cf)/ M] ×V (Fan et al., 2008)

Where Ci and Cf are the initial and residual Zn(II) concentrations (mg/l), respectively. M is the biosorbent mass (g), V is the volume of the solution, and q is the sorption capacity (mg/g). The native and alkali-treated biomass before and after Zn(II) uptake was investigated by EDX and FTIR. Biomass investigated with Perkin-Elmer FTIR 1650 at the Center of Microanalysis, Cairo University, Cairo, Egypt.

### 2.9 Statistical analysis

All the experiments were conducted in biological triplicates and the results were expressed by the mean ± STDEV. The significance was calculated with one-ANOVA with Fisher's Least Significant Difference of post hoc test.

## 3 Results and discussion

### 3.1 Zinc tolerance and its effect on the growth of *F. solani*

Metal resistance is the ability of microorganisms to withstand heavy metals toxicity through one or more mechanisms designed to respond directly to the metals involved (Iram et al., 2013). The utilization of mycoremediation to minimize metal pollution is based on the tolerance and bioaccumulation capacity of a specific fungus (Di Piazza et al., 2018). *F. solani* displayed tolerance to Zn(II) up to 10000 mg/l. Average daily Zn(II) intake from drinking-water is should be less than 0.2 mg/day (WHO, 2001). Patchy irregular growth of *F. solani* was observed at >8000 mg of Zn(II)/l. Yazdani et al. (2010) reported that *T. atroviride* was highly tolerant of Zn(II) and can grow at 6000 mg/l. When assessing the TI, *F. solani* showed a very high tolerance at 1000 mg/l (TI = 1.00), high tolerance at 2000–4000 mg/l (TI = 0.99 and 0.88, respectively), moderate tolerance at 6000 mg/l (TI = 0.61), low tolerance at 8000 mg/l (TI = 0.50) and very low tolerance at 9000 mg/l Zn(II) (TI = 0.17). Low Zn(II) concentrations increased the growth of eight litter-decomposing basidiomycetous fungi by 2%–272%. In contrast, high Zn(II) concentrations completely inhibited the fungal growth (Hartikainen et al., 2012).

### 3.2 SEM, TEM, and EDX investigations

**Figure 1:**
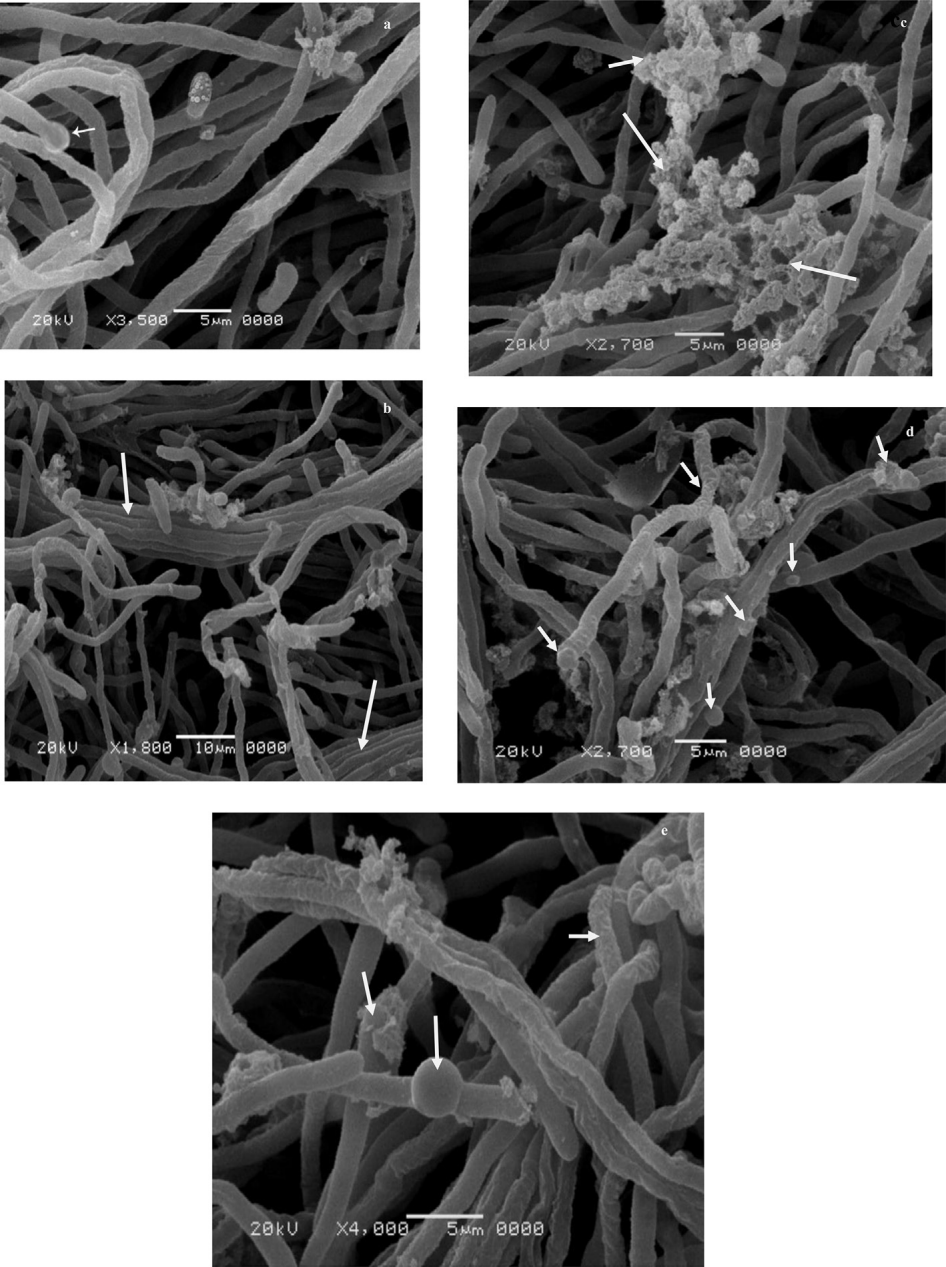
SEM micrograph of growing *F. solani* (a) Zn(II)-free pellets (control), (b–e) Zn(II)-loaded pellets (9000 mg/l). *F. solani* cultures were incubated for 7 days at 28 °C.

**Figure 2:**
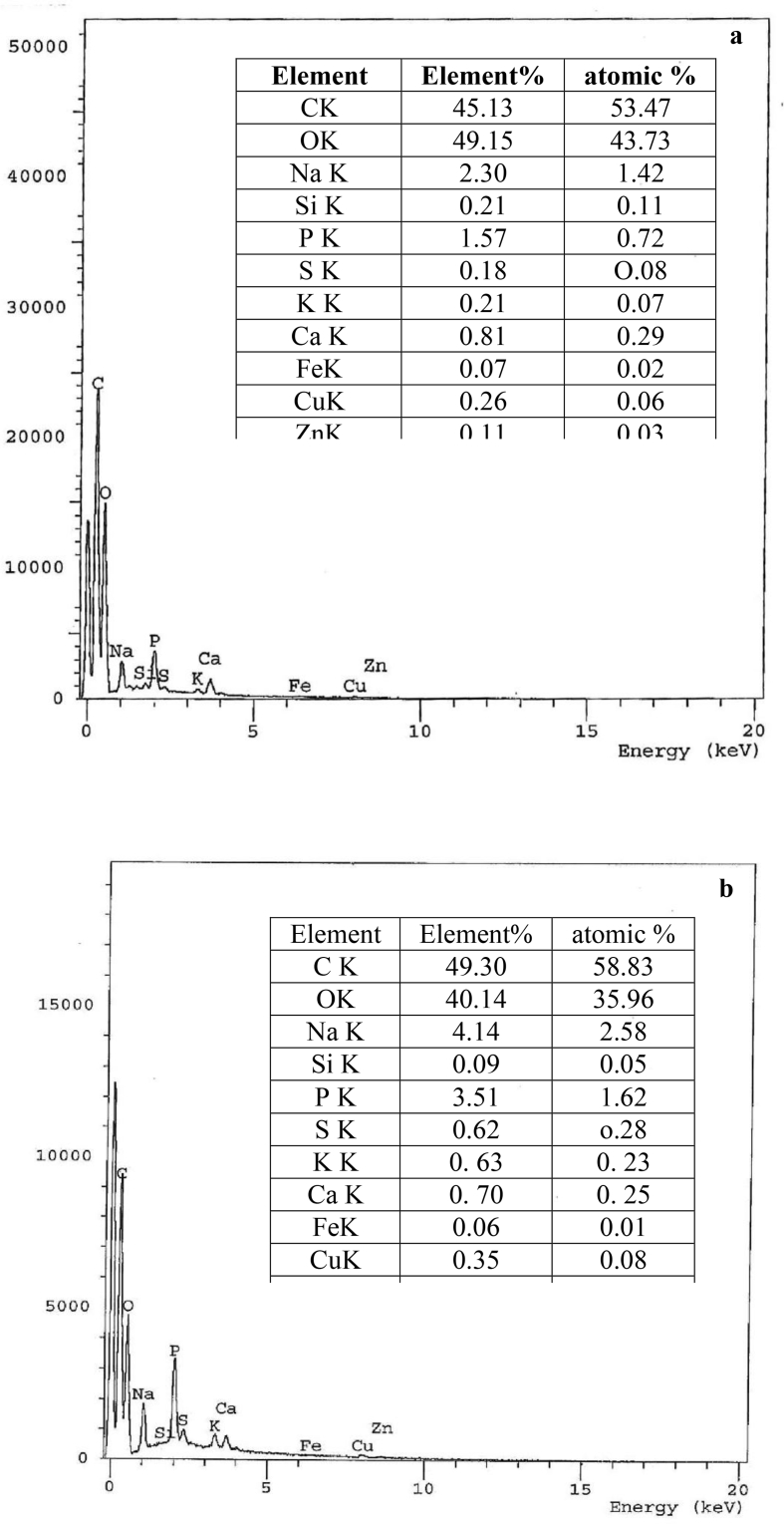
EDX of growing *F. solani* (a) Zn(II)-free pellets (control), Zn(II)-loaded pellets (9000 mg/l). *F. solani* cultures were incubated for 7 days at 28 °C.

To recognize the effect of metal on the biomass surface during Zn(II) bioaccumulation, mycelia of *F. solani* subjected to SEM (Figure 1a–e) and EDX examinations (Figure 2 a and b). The surface of the mycelia was smooth before exposure to Zn(II) (Figure 1a). As shown in Figure 1b, curling; and formation of mycelia clusters in response to Zn(II) stress was observed. Moreover, the mycelia became covered by a substance that could be a precipitate-containing Zn(II) (Figure 1c). The surface of *F. solani* also had a rough texture with the formation of protrusions on the hyphae (Figure 1d, e). The gathering of mycelia and formation of coils could be likely due to the excretion of polysaccharides as a fungal resistance mechanism (Wan Maznah et al., 2012). The adaptation of fungi to metal stress caused the modifications of the cell surface that depended on the type and concentration of metal and thought to be associated with intracellular detoxification of heavy metals (Kim et al., 2012; Luna et al., 2015). These changes refers to the formation of intracellular vacuoles that act as storage compartments for thiol-containing compounds that can bind metal ions and accumulate them in the vacuoles and hence increase the pressure within the mycelia leading to cell wall protrusions (Paraszkiewicz et al., 2010, Li et al., 2017, Gururajan and Belur, 2018, El-Sayed et al., 2020 a,b).

The EDX microanalysis is a valuable tool focused on the production of distinctive X-rays showing semi-quantitative as well as semi-qualitative element data in the samples (Siddiquee et al., 2015). The EDX spectrum of control biomass revealed a very weak signal for Zn(II) (Figure 2a). The 3.18-fold rise of Zn(II) relative to control referred to metal adsorption on the surface of *F. solani*. There was a 1.8, 2.24, 3.44, 3.15, and 1.7 folds increase in element % of Na, P, S, K, and Cu, respectively (Figure 2b), that could be due to the participation of ions and complexation during the bioaccumulation process. Treatment of fungi with high doses of metal ions caused an increase in cysteine synthesis and release of phosphorus. Phosphorus and sulfur could sequester and chelate excess metal ions (Lima et al., 2013).

**Figure 3:**
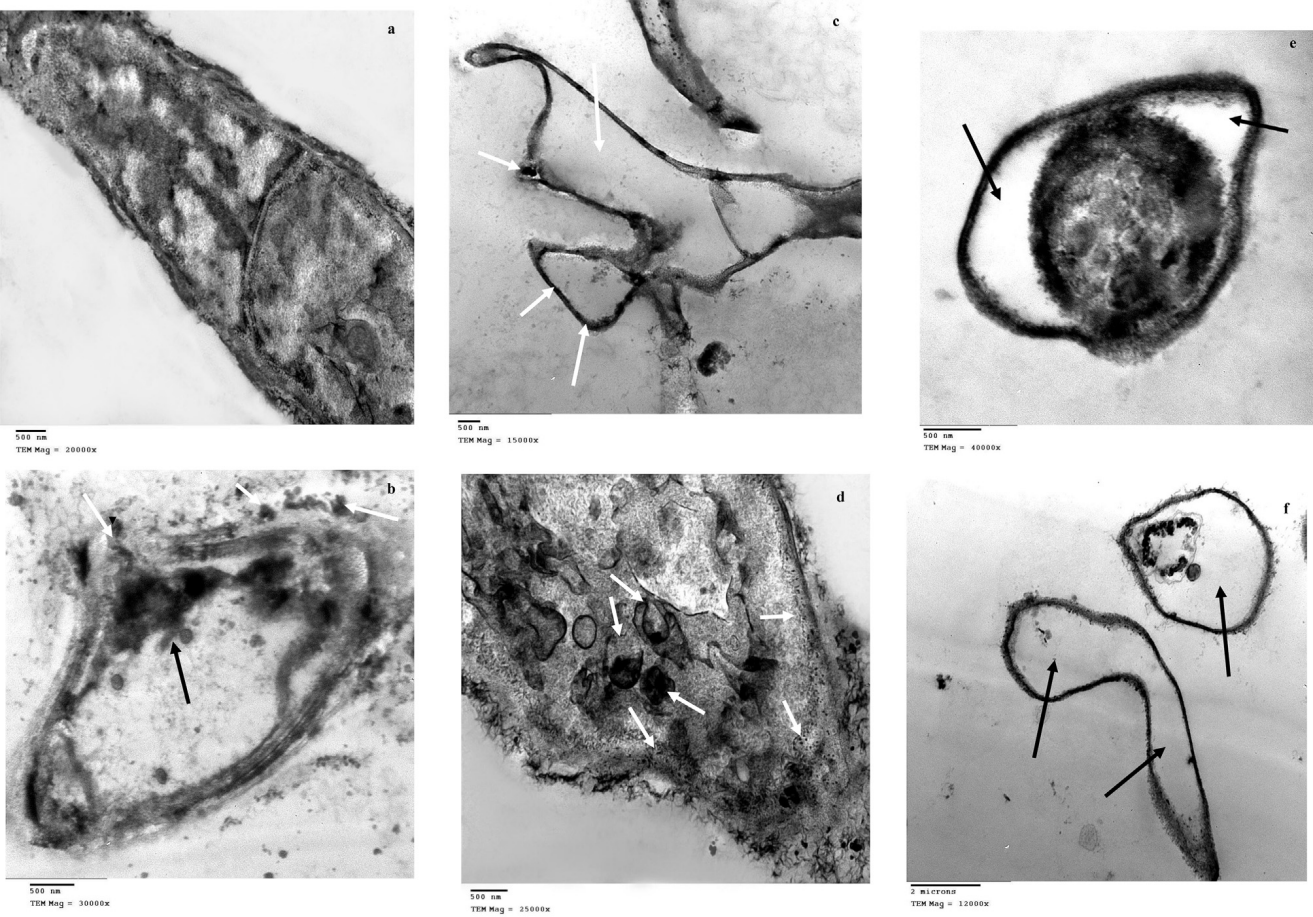
TEM of growing *F. solani* (a) Zn(II)-free pellets (control), (b–f) Zn(II)-loaded pellets (9000 mg/l). *F. solani* cultures were incubated for 7 days at 28 °C.

Transmission electron microscopy was used to assess the mechanism of Zn(II) remediation (Figure 3a–f). TEM micrographs of metal-unloaded cells displayed a complete cell wall (170 nm in thickness) and homogeneous cytoplasm with few electron-dense granules probably clarify the cytoplasmic deposits and genetic materials (Figure 3a). Zn (II)-stressed cells showed a ruptured wall with exclusion of some cellular contents, and formation of intra- and extracellular precipitates suggesting the homogenous Zn(II) compatibilization (Figure 3b). The precipitation outside the cell seemed to be the first defense of *F. solani* against Zn(II). The biosorption of heavy metals depended on ionic species associating with the cell surface or extracellular polysaccharides, proteins, and chitins (Garcıa-Hernandez et al., 2017). The complete lysis of cytoplasmic organelles and the formation of some precipitates within the dark cell wall was observed (Figure 3c) and this distortion may be due to the oxidative stress of Zn(II). The tolerance and ability to detoxify metal ions have been addressed like valence transformation, extra and intracellular precipitation and active uptake (Siddiquee et al., 2015). Sequestration within vacuoles, and formation of nanoparticles within the periplasm space (Figure 3d). Zn(II)-loaded cells had relatively thinner (90 nm) and darker cell walls. Plasmolysis and lysis of internal organelles were observed (Figure 3e and f). Metal immobilization includes vacuoles compartmentation and complexation by cytoplasmic protein (Gonzàlez-Guerrero et al., 2008). Fungi can facilitate biotransformation of metals by chemical reactions like methylation, oxidation, reduction, and dealkylation, that reduce metal toxicity (Saha and Orvig, 2010).

### 3.3 Effect of Zn(II) on *F. solani* growth

The dry weight was increased by about 10.21% comparing to Zn(II)-free media at 200 mg/l of Zn(II), with the increasing on the initial concentration of metal from 500 to 6000 mg/l, while the fungal growth was decreased by 84.2 % at 7000 mg/l of Zn(II) (Figure 1s). When the initial Zn(II) concentrations increased from 200 to 4000 mg/l, the removal efficiency was increased from 28.5 to 94.9 % (Figure 2s). The microbial growth on solid media did not give a correct picture of metal tolerance where the complexation, diffusion, and availability of metals differ from those in the broth, agar had protecting effects and chelate metal ions. In consequence, the heavy metals became slightly available for the growth, giving a miss-indication to a higher tolerance response (Moghannem et al., 2015), therefore, tolerance assay compared in both solid and liquid media.

Simultaneously, a decrease in the uptake of Zn(II) by about 32.5% was occurred with 6000 mg/l, the metal uptake relies on availability of active sites present on the surface of biomass and metal concentration. As long as the active sites are free, the specific metal removal was increases with the higher Zn(II) concentration (Sharma et al., 2002). Bioaccumulation comprises the incorporation of many processes such as complexation, electrostatic attraction, covalent binding, ion exchange, van der Waals forces, precipitation, and adsorption (Vaishaly et al., 2015). Uptake of metal ions by fungi has been stated to involve an initial rapid binding of metal ions to negative functional cell wall groups, such as amide, carboxyl, phosphate, hydroxyl, and sulfhydryl followed by a slower energy-dependent entry (Cecchi et al., 2017a).

**Figure 4:**
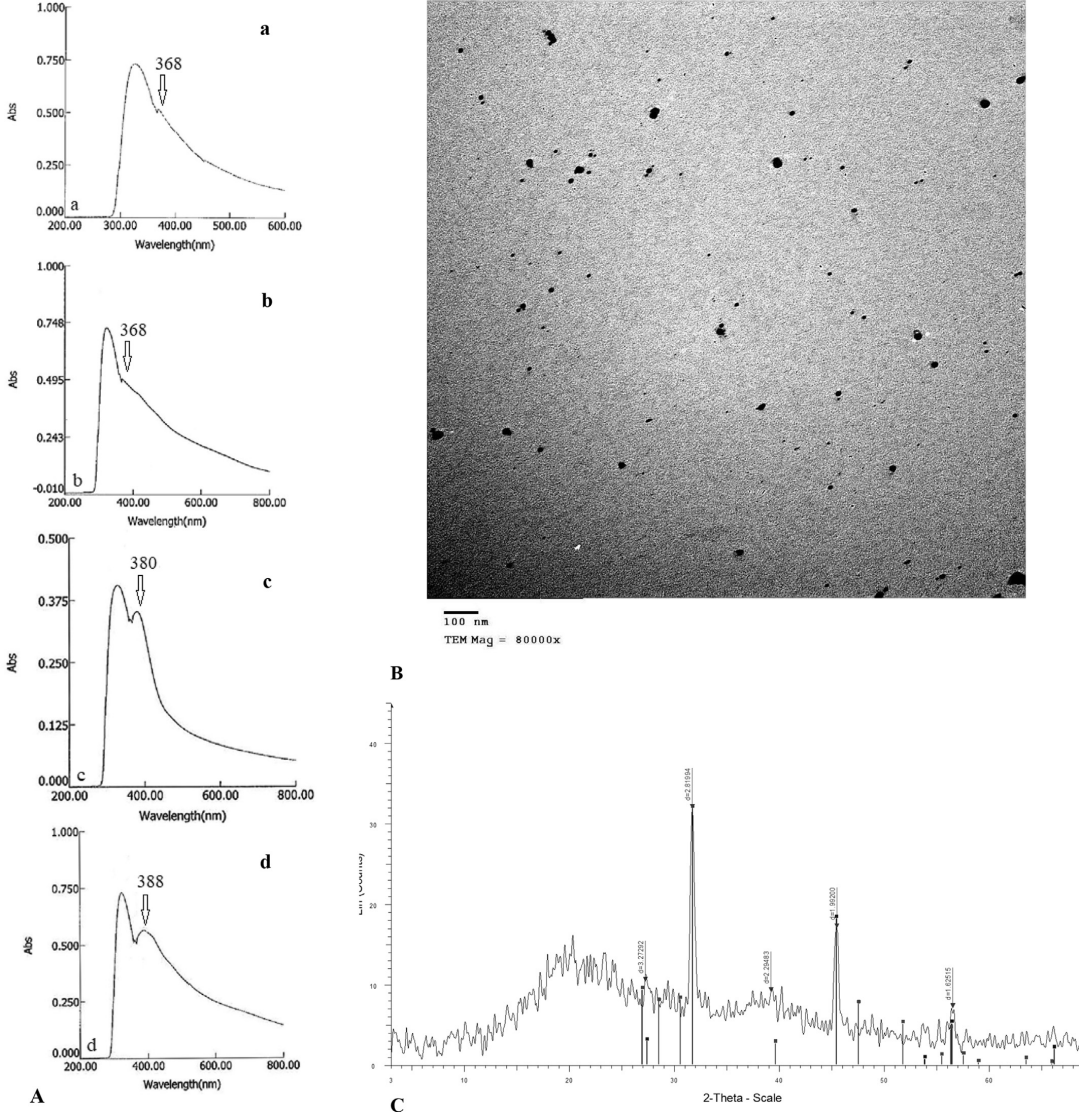
A, UV-Visible spectra of zinc oxide nanoparticles synthesized by *F. solani* in the presence of (a) 500 mg Zn(II)/l, (b) 1000 mg Zn(II)/l, (c) 4000 mg Zn(II)/l, and (d) 5000 mg Zn(II)/l. B, TEM image of zinc oxide nanoparticles synthesized by *F. solani* in the presence of 1000 mg Zn(II)/l. C, XRD pattern of zinc oxide nanoparticles synthesized by *F. solani* in the presence of 1000 mg Zn(II)/l.

The recognition of ZnONPs was achieved by creating white coalescence at ≥500 mg/l. The Surface Plasmon Resonance (SPR) peaks were observed at 368 nm (500 mg/l Zn(II)), 368 nm (1000 mg/l Zn(II)), 380 nm (4000 mg/l Zn(II)) and 388 nm (5000 mg//l Zn(II))) (Figure 4A). The concentration of ZnONPs was 300 mg/l as determined by UV-analysis. The position of SPR peaks relies on the particle shape, size, and adsorption of electrophile or nucleophile to surface of the particle (Umadevi et al., 2012). The diameter of spherical ZnONPs was extended from 19.67 to 32.12 nm (25.22 ± 7.14 nm) (Figure 4B). The particles were confirmed as elemental Zn(0) by XRD (Figure 4C). Oxidoreductase implementation could be among the detoxification mechanisms (Iravani, 2011).

### 3.4 Catalase and polyphenol oxidase activity

In response to heavy metal, an uncontrolled synthesis of reactive oxygen species (ROS) have been reported. Damaging influences of ROS to cellular constituents diminished by antioxidant defense mechanisms including enzymatic mechanisms that based mainly on superoxide dismutase, catalase, polyphenol oxidase and glutathione *S*-transferase have been observed (Hu et al., 2015). The CAT activity was slightly induced (8.31%) by the growth in 200 mg/l of Zn(II)-enriched media and reached its highest value at 2000 mg/l. Compared to control, the PPO activity enhanced by 246.8% at 4000 mg/l Zn(II) (Figure 3s). Under metal stress, the level of ROS in the cells surpassed the tolerance level of natural antioxidant systems (Bai et al., 2015). Due to toxic metal stress, the activities of antioxidant enzymes could be changed by these ways: 1) a regular increase in enzymes activities with the increase on metal concentration. 2) an increase in the activities of enzymes to attain the highest values and then declined with a further increase in metal concentration (Kusvuran et al., 2016). However, according to the present findings, the changes in PPO and CAT activities belonged to the second type. Similar results reported by Feng et al. (2018).

### 3.5 Total antioxidants, thiols contents, soluble protein content of *F. solani*

**Figure 5:**
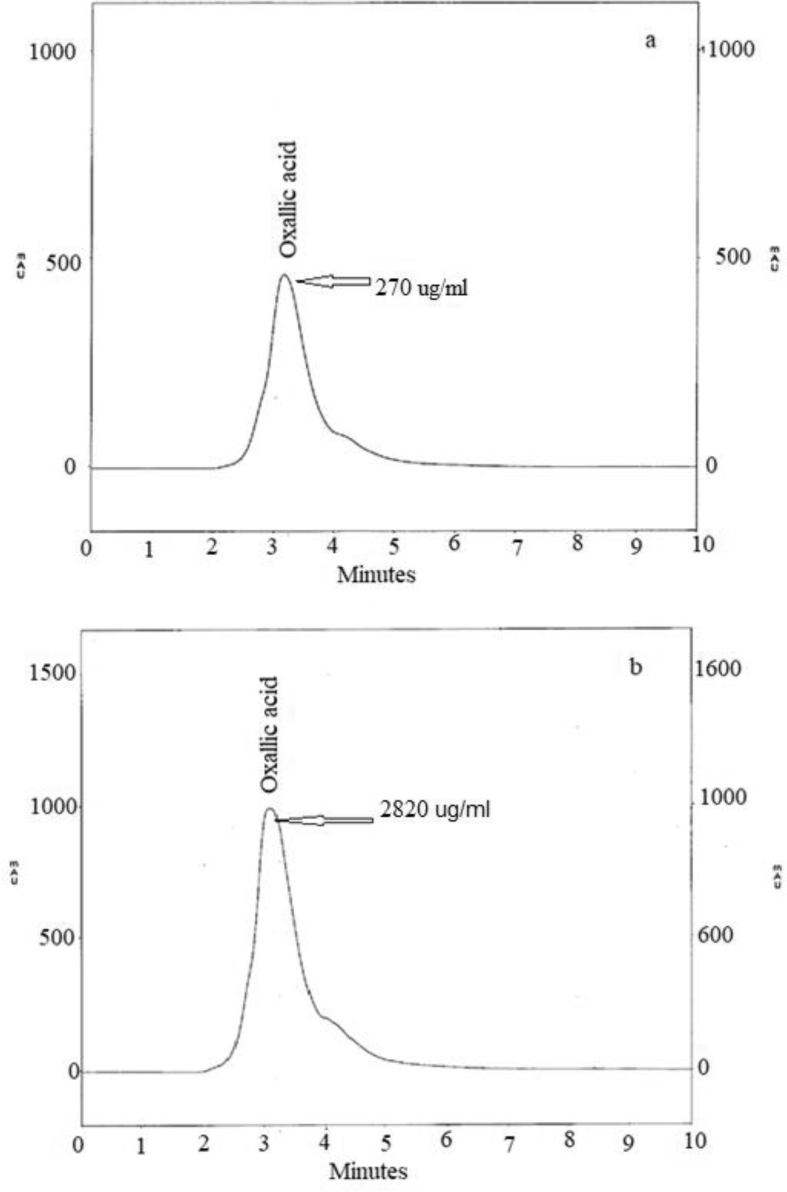
HPLC chromatograms of *F. solani* (a) Zn(II)-free culture filtrate (control), (b) Zn(II)-supplemented culture filtrate (4000 mg/l).

**Figure 6:**
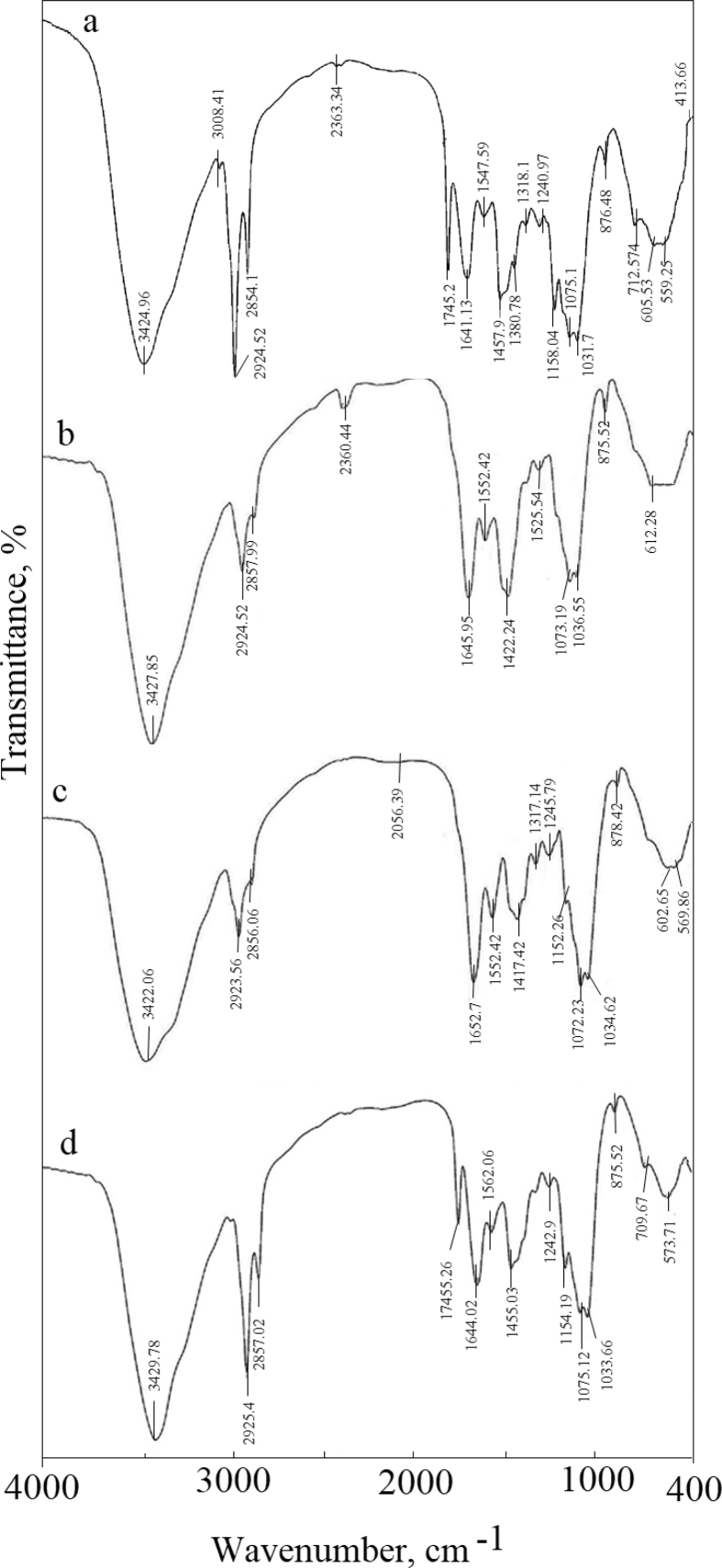
FTIR spectra of *F. solani*, (a) native cells, (b) Zn(II)-loaded cells, (c) alkali-treated biomass, and (d) Zn(II)-loaded alkali treated cells. Biosorption conditions: initial pH = 4 (alkali-treated biomass) and 5.0 (native biomass), initial Zn(II) concentration = 600 mg/l, biosorbent dose = 1.0 g/l, contact time 40 min (alkali-treated biomass) and 6 h (native biomass), temperature = 30 °C at 140 rpm.

A linear increase on the total antioxidants was found in response to increasing concentrations of Zn(II) (Figure 4s). The total antioxidants could be an organic acids, phenolic compounds, amino acids, vitamins and some metallic ions. A plateau region was noticed at 200 and 500 mg/l. A noticeable increase in extracellular total antioxidants (that present on the fungal filtrate) (37.62%, relative to control) and intracellular (112.67%) was noticed at 4000 mg/l. This would reveal a remarkable effort that was done by *F. solani* to reduce the excess toxicity by mobilizing non-enzymatic antioxidants to trap excess Zn(II) and remove it outside the cell. The highest intracellular and extracellular thiols (115.3 and 92.3 mM/g, respectively) was recorded at 1000 mg/l. Thiol contents was drastically decreased at 2000 mg/l and inhibited at 4000 mg/l (Figure 5s). Thiols could be involved in metal homeostasis and metal detoxification (Kalsotra et al., 2018). The decrease in thiol contents at 2000 mg/L Zn(II) showed the inability of *F. solani* to tolerate such stress and disturbances induced by high Zn(II) concentrations in cellular tolerance/detoxification mechanism (Mukherjee et al., 2010). The present results showed that the intra and extracellular soluble protein reached the maximum values at 2000 mg/l and 4000 mg/l concentration of Zn(II) (43.37% and 117.97%, respectively, compared to control) (Figure 6s). These proteins (metal tolerance/transport) could be involved in helping the cells to preclude unnecessary amounts of metal ions from the cytoplasm (Koźmińska et al., 2018). A decline in total soluble protein observed at higher concentrations of Zn(II).

### 3.6 Lipid peroxidation and H_2_O_2_ content

**Figure 7:**
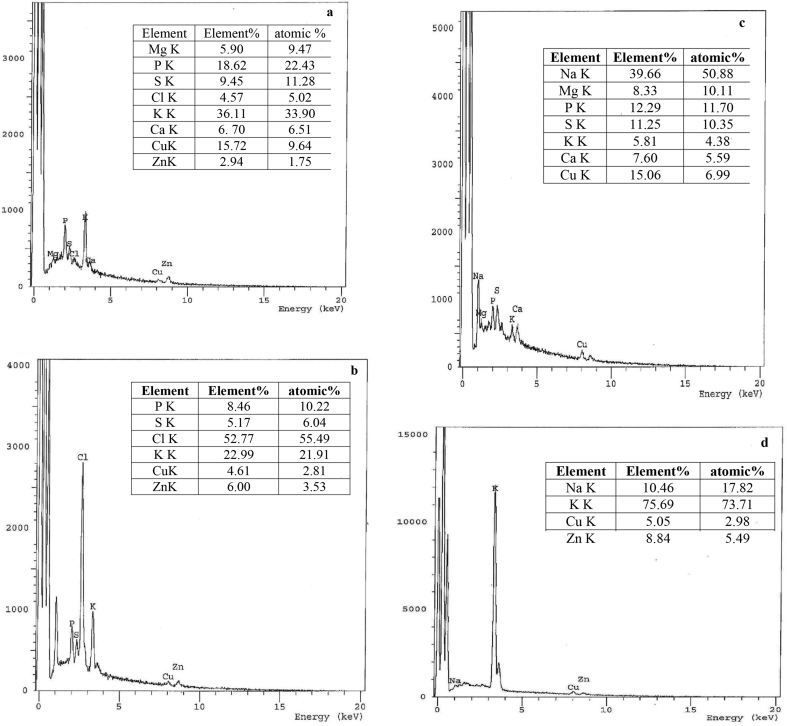
EDX microanalysis of *F. solani* (a) native cells, (b) Zn(II)-loaded cells and (c) alkali-treated cells, and (d) Zn(II)-loaded treated cells. Biosorption conditions: initial pH = 4 (alkali-treated biomass) and 5.0 (native biomass), initial Zn(II) concentration = 600 mg/l, biosorbent dose = 1.0 g/l, contact time 40 min (alkali-treated biomass) and 6 h (native biomass), temperature = 30 °C at 140 rpm.

ROS reaction with methylene groups of the polyunsaturated fatty acids causes lipid peroxidation, releasing malondialdehyde (MDA) as one of the terminal by-products. MDA values usually reflect the level of damage to plasma membranes (Hu et al., 2015). *F. solani* exposed to ≤500 mg/l showed no accumulation of MDA, while, exposure up to 4000 mg/l, led to an increase in MDA content accompanied by a plateau up to 6000 mg/l. H2O2 content was increased with Zn(II) treatments but decreased markedly at 6000 mg/l (Figure 7s). The substantial increment in MDA titer revealed the elevated formation of ROS (Mukherjee et al., 2010).

### 3.7 Oxalic acid secretion

Oxalic acid produced by some fungal isolates are mainly used to immobilize potentially toxic metals by forming insoluble compounds such as complex of metal-oxalate (Siddiquee et al., 2015). From the HPLC analyses, the Zn(II)-free and Zn(II)-stressed samples displayed an oxalic acid concentrations 270 and 2820 μg/ml, respectively (Figure 5a and b). Zn(II) stimulated oxalic acid production by about 10.5 folds, comparing to control. It plays a prominent role in the tolerance of fungal consortia contained *Aspergillus niger*, *Penicillium* sp., and *Rhizopus* sp. to Cu(II) and Pb(I) (Shivakumar et al., 2014).

### 3.8 Initial pH

The initial pH seemed to be a substantial factor influencing the biosorption process. It determines the solution chemistry and complexation of the ions (Frutos et al., 2016). Furthermore, it influences the natures of biomass and binding sites activities. Biomass regarded as natural ion-exchange materials that chiefly have positively and negatively charged groups. The Zn(I)) removal capacities of native and alkali-treated biomass were low at pH 2.0 (2.5 and 4.21 mg/g, respectively) (Fig 8s). When pH is less than 3, poor ionization or protonation of functional groups cause a weak complexation affinity between ions and cell wall (Iram et al., 2015). When pH increased to 5 (native biomass) and 4 (treated biomass), the removal capacities was enhanced to 136.4% and 178.69%, respectively. As the pH increased [H3O]^+^ levels was decreased and the sites were deprotonated. Therefore the competitive effects of hydronium ions were limited, and the exchange of protons with Zn(II) preferred (Mrudula et al., 2016).

The subsequent decline in biosorption ability was due to ions speciation and their precipitation as metal hydroxides (Hlihor et al., 2014). Furthermore, the degree of ionization of organic molecular groups and the release of organic ligands from the cells increased at high pH. The ligands made soluble complexes with the ions and diminished the biosorption capacity. *Pleurotus* spp. had optimum biosorption capacities of Ni(II) and Cu(II) between pH 5 and 6 (Tay et al., 2012). The alkali treatment enhanced the biomass electronegativity by ionizing the functional groups and hence attracting many cations (Bux and Kasan, 1994). Wang and Chen (2006) suggested that the deacetylation of the fungal cells affected the chitin structure and led to the formation of chitosan-glycan complexes and improved metal affinities.

### 3.9 Initial metal ion concentration

Native and treated mycelia's sorption abilities exponentially increased (from 2.79 to 7.1 mg/g, and 5.81–12.5 mg/g, respectively) with increased concentration of Zn(II) from 200 to 600 mg/l (Figure 9s). Nevertheless, a further rise in the metal concentrations to 700 mg/l resulted in a decline in Zn(II) biosorption to 6.3 mg/g (native biomass) and 10.1 mg/g (treated biomass), suggesting overload of all binding sites and a balance between biosorbents and adsorbents. The concentration of metal ions played a significant role as a driving potential in overcoming the resistance to mass transfer between solid and aqueous cases. Bio-removal increases with an increase in initial concentration at certain biomass dose (Abbas et al., 2014). At lower initial concentration, the amount of the initial moles solute to the accessible surface area was the minimum. Because of such, the fractional biosorption did not rely on the initial metal concentration (Binupriya et al., 2007). Maximum capacity at 600 mg/l Zn(II) associated with the higher mass transfer and kinetic energy, and availability of metal ions thus the possibility of collision between the biosorbent and the ions (El-Gendy et al., 2017). Reducing the biosorption capacity at higher concentrations could be ascribed to the inadequacy of free accessible binding sites and the competition between ions (Garcıa-Herna´ndezet al. 2017).

### 3.10 Biosorbent concentration

The adsorbent concentrations played a key role in the uptake due to the durable dependency on the number of available sites and the electrostatic interactions between biosorbent cells (Shamim, 2018). The uptake capacities are inversely proportional to the biomass doses. The highest capacities of uptake of native (6.84 mg/g) and treated biomass (12.8 mg/g) was achieved at the biosorbent dose 1.0 g/l (Figure 10s). At a given equilibrium, the biomass adsorbs more metal ions at low cell densities than at high densities. The uptake capacities progressively decreased with a further increase in the biosorbent concentrations and reached the lowest values (2.1 mg/g, native biomass and 4.57 mg/g, treated biomass) at 5 g biosorbent/l. High biomass concentrations can exert a shell effect that restricts the access of metal ions to binding sites (Kanamarlapudi et al., 2018). Moreover, at the higher biomass dosage, the metal ions are not enough for complete distribution over the accessible binding sites.

### 3.11 Effect of temperature

The temperature has an important impact on the biosorption as it can make chemical moieties ionization and influences the cell wall's firmness and its structure (Iram et al., 2015). There was a gradual increase in the metal uptake with a rise in temperature (from 10 to 40 °C) reaching a maximum of 7.7 mg/g (native) and 13.2 mg/g (treated) (Figure 11s) at 30 °C. However, the biosorption capacity reduced by 76.62% (native) and 73.48% (treated) at 60 °C. As the collision frequency between *F. solani* and Zn (II) increased at 30 °C, more zinc particles electrostatically sorbed on the biosorbent. It is usually supposed that the biosorption process is carried out between 20 and 35 °C. Temperatures above 45 °C may result in the structural damage to proteins which in turn impacts metal uptake (Deng and Wang, 2012). *A. flavus* and *A. niger* exhibited maximum sorption capacity for Cu(II) at 26 °C and 37 °C, respectively (Iram et al., 2015).

### 3.12 Effect of contact time

Time-course profiles for Zn(II) uptake by *F. solani* showed that the saturation levels reached within 40 min (treated biomass, q = 12.5 mg/g) and 6 h (native biomass, q = 7.9 mg/g) (Figure 12s). The plateau levels accomplished within 2 h and 12 h for treated and native biomass, respectively. Then, Zn(II) uptake slightly declined after 12 h. The results proved two stages of the process, a rapid initial one assigned to the surface adsorption. The subsequent slow phase ascribed to membrane transport into the cell or reduced cell wall permeability or slow intracellular diffusion (Shamim, 2018). The time needed to achieve maximum uptake depends on type of biosorbents, metals, and their interactions (Kanamarlapudi et al., 2018; Chatterjee et al., 2010).

### 3.13 Surface characterization

#### 3.13.1 FTIR

Fungal cell walls composed of complicated macromolecules like chitins, mannans, proteins, glucans, lipids, and pigments, such as melanins. In general, polysaccharides are the main components and constitute about 90% of the wall. Various types of ionizable sites influence metal absorption capacity: COOH^−^ (carboxyl groups), and –OH (hydroxyl groups) on uronic acids and proteins, –SH (sulfhydryl groups), and nitrogen-containing ligands on proteins, chitin, and chitosan, and (PO4)^3-^ (phosphate groups) (Shamim, 2018). The FTIR spectra of NU (native-unloaded biomass), NL (native-loaded biomass), TU (treated-unloaded biomass), and TL (treated-loaded biomass) depicted in Figure 6a–d, respectively. The shift in the wave number at 3424.96 cm^−1^ (NL) (Figure 6b) and 3429.78 cm^−1^(TL) (Figure 6d) assigned to the interaction of –NH_2_ asymmetric stretching mode of amines and –OH groups with Zn(II) uptake (Gururajan and Belur, 2018). The disappearance of 3008.4 cm^−1^(NL) peak was due to C–H stretching frequencies (Bright et al., 2010). Changes in the peak intensity at 2924.52 and 2854.14 cm^−1^(NU) and 2925.48 cm^−1^and 2857.02 cm^−1^(TL) can be attributed to CH_3_ symmetric stretching of proteins and lipids and CH2 symmetric stretching, respectively (Zhang et al., 2015). The shift at 2363.34 cm^−1^with an increase in the intensity (NL) was due to the asymmetric stretching of the –N=C=O- group (Mishra and Jha, 2009). The new band at 2065.39 cm^−1^, the disappearance of 1745 cm^−1^(NL and TL) peak, and shift at 1641.13 cm^−1^(NL) and 1644.02 cm^−1^(TL) are due to the C=O stretching mode of the carbonyl group in esters, alcohol, and carboxylic acids. The noticeable shift at 1455.2 to 1417.4 cm^−1^(TL) and 1422.24 cm^−1^(NL) was due to the C–N stretching, N–H bending vibration, and complexation with N–H group (Hu et al., 2015). The shift also indicating the acidic groups; carboxyl and hydroxyl, are chief agents in uptake (El-Gendyet al. 2017). The shifts at 1547.59 cm^−1^(Δ 5 cm^−1^, NL) and 1562.06 cm^−1^(Δ10 cm^−1^, TL) attributed to N–H bending strongly coupled with C–N stretching (amide II band) (Feng et al., 2018). A marked shift at 1457.59 cm^−1^(Δ 35 cm^−1^, NU) and 1455.03 cm^−1^(Δ38 cm^−1^, TL) was assigned to CH3 asymmetric bending vibration of protein (Ramalingam et al., 2014). The role of amide III, sulfonamide, and C(O)–O stretching vibrations recognized in the disappearance of peaks at 1380.78 and 1318.11 cm^−1^(NU) and a new peak at 1317.14 cm^−1^(TL). The shift at 1240.97cm^−1^was due to P=O asymmetric stretching of phosphodiesters in phospholipids. The disappearance of the peak at 1158.04 cm^−1^ (NL) assigned to stretching of C–O. The shift at1075.12 cm^−1^ indicated the Zn(II) interaction with sulfoxides, S=O stretching, sulfones, and sulfonic acid. The shift at 1031.73 cm^−1^(NL) is due to the binding of heavy metals to phosphate groups (Mahmoud et al., 2011). El-Gendy et al. (2017) reported the binding of phosphorus compounds, C–N stretching, O–H bending, and sulfur compounds in the region 1000–1400 cm^−1^. A very marked shift at 573.72 cm^−1^(Δ 29 cm^−1^) (TL) and a new peak at 559.26 cm^−1^(NL) revealing the C–S stretching. The absence of peaks at 712.57 cm^−1^(NL) and 709.68 cm^−1^(TL) and the change in intensity of peaks at 875 cm^−1^assigned to N–H wag of primary amines (Mishra and Jha, 2009). The C–S stretching reveals the appearance of a new band at 413.66 cm^−1^in the case of NL biomass. Similar results have been reported for soft metals that form stable bonds with sulfur-containing (soft) ligands, nitrogen-, S-, SH-, CN-, R–NH_2_−, and imidazole (Wang and Chen, 2006). The higher covalent index (X2mr) (Xm is electronegativity and r is the ionic radius), the greater the potential to form covalent bonds with biological ligands in order S > N > 0 (Chen and Wang, 2007). The electronegativity and the ionic radius of Zn(II) are1.65 and 139 pm, respectively. The covalent index of Zn(II) is 3.78. After Zn(II) uptake, the total shifts in TL biomass (Δ 100cm^−1^) were more pronounced than in NL (Δ 75 cm^−1^). C–S stretching was involved in the process by TL than NL biomass. CH_3_ asymmetric bending vibration of proteins was involved equally. After Zn(II) biosorption, the intensities of all peaks was increased in the case of NL while decreased in TL.

#### 3.13.2 Energy dispersive X-ray (EDX) microanalysis

EDX analysis was used to confirm the identity of Zn(II) on the fungal cell surface. The EDX spectra of NL (Figure 7b) and TL biomass (Figure 7d) were marked by the appearance of Zn(II) by 6.00 and 8.84 element%, respectively. Simultaneously, P and S signals disappeared after Zn(II) uptake by TL biomass. The element% of P and S was reduced by 54.56 and 45.29%, respectively, after Zn(II) uptake by NL biomass. It was reasonable to conclude that some sulfur and phosphorus organics were released from the cells to the supernatant during Zn(II) uptake. Similarly, Na(I), Mg(II), K(I), Ca(II), and Cu(II) was released during biosorption. Usually, the release of these metal ions from biosorbents in binding Zn(II) was regarded as an indicator of the mechanism of ion exchange for heavy metal binding (Ali et al., 2016; Patel et al., 2016; Reddad et al., 2002). Similar results reported by Can and Jianlong (2008). They concluded that ion exchange of K(I), Mg(II), Na(I), or Ca(II) with Zn(II) during biosorption by *Saccharomyces cerevisiae* indicated a certain degree of the ionic binding interaction between Zn(II) and the biomass.

## 4 Conclusion

Fungi are one of the most applicable microorganisms for remediation of toxic heavy metals due to their powerful biosorption and biotransformation potency, nevertheless, few studies uncovering the mechanisms of fungal removal of heavy metals have been reported. The pattern of growth, bioaccumulation, organic acids production, non-proteineous antioxidants, and antioxidative enzymes of *F. solani* responsive to Zn(II) were determined. It has been observed that oxalic acid of *F. solani* was increased by 10 folds due to the presence of Zn(II) regarding to the control. The ratio of Zn(II) ions biosorption is strongly dependent on the treated biomass, pH values, initial metal ion does, incubation temperature, and time of contact.

## Declarations

### Author contribution statement

Manal T. El Sayed: Conceived and designed the experiments; Performed the experiments; Wrote the paper.

Ashraf S.A. El-Sayed: Analyzed and interpreted the data; Wrote the paper.

### Funding statement

This research did not receive any specific grant from funding agencies in the public, commercial, or not-for-profit sectors.

### Competing interest statement

The authors declare no conflict of interest.

### Additional information

No additional information is available for this paper.

## Appendix A Supplementary data

Supplementary_Data_Zinc Supplementary_Data_Zinc
