# Supplementary material for: Bioremediation and tolerance of zinc ions using Fusarium solani
Source: Heliyon. 2020 Sep 28;6(9):e05048. doi: 10.1016/j.heliyon.2020.e05048 (PMC7527588; doi:10.1016/j.heliyon.2020.e05048)
Supplement: Supplementary_Data_Zinc [file mmc1.doc]

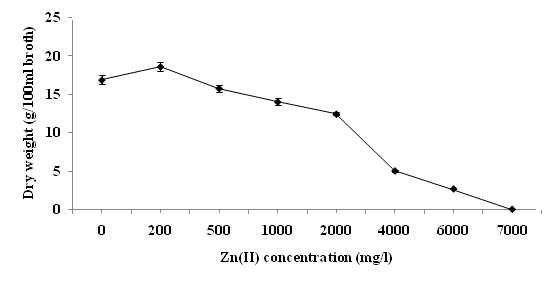


**Fig. 1s:** Effect of different concentrations of Zn(II) (mg/l) on dry weight of *F. solani*. Data represent means of 3 measurements ±SE


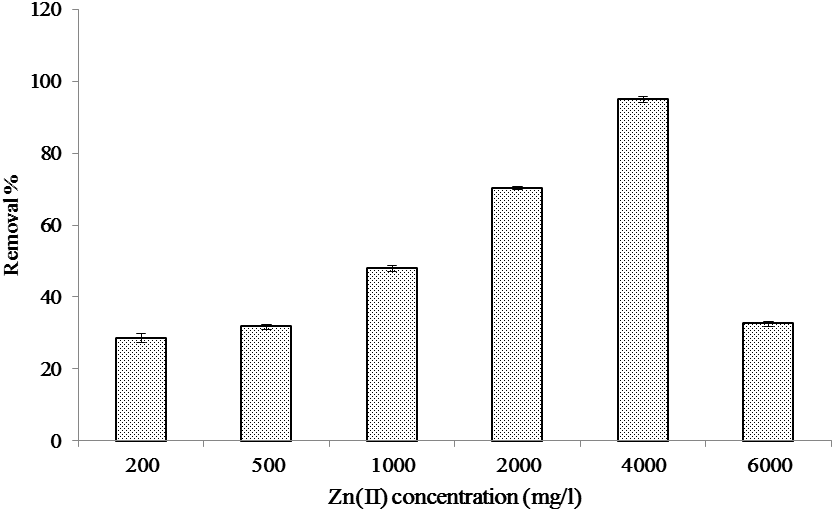


**Fig. 2s:** Effect of different concentrations of Zn(II ) (mg/l) on the removal % of *F. solani*. Data represent means of 3 measurements ±SE


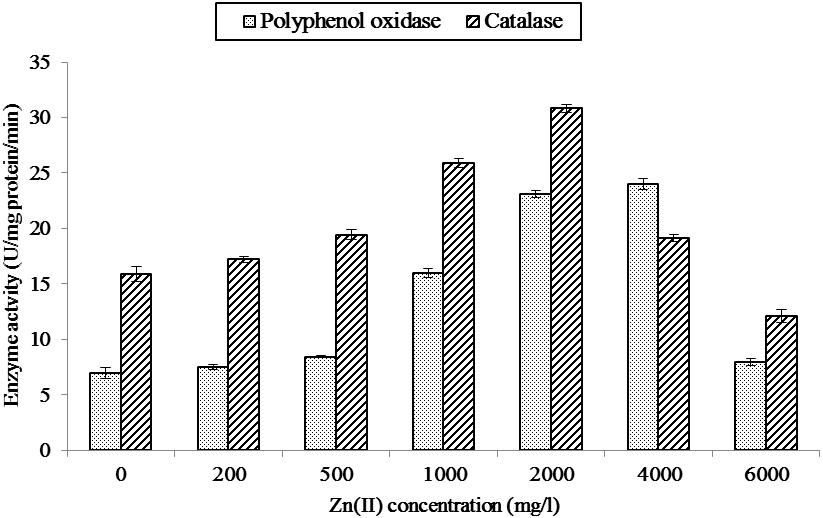


**Fig. 3s:** polyphenol oxidase and catalase activity in *F. solani* exposed to different concentrations of Zn(II). Data represent means of 3 measurements ±SE


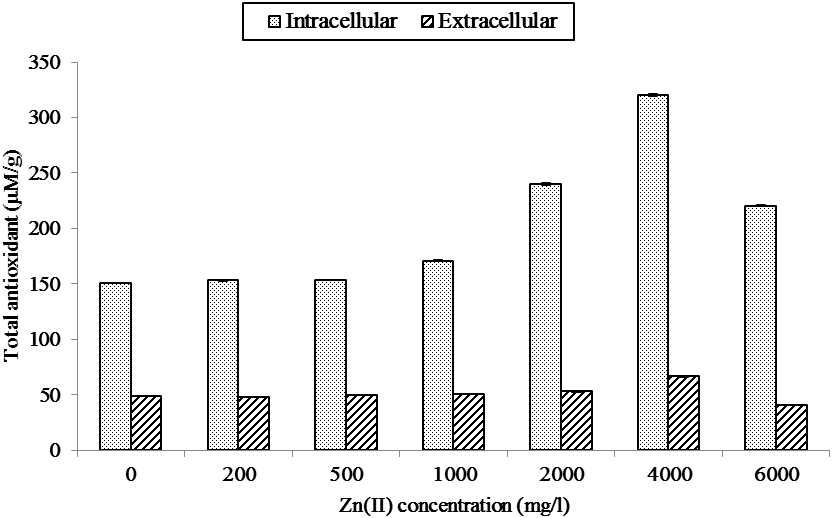


**Fig. 4s:** Extracellular and intracellular total antioxidants in *F. solani* under different Zn(II) concentrations. Data represent means of 3 measurements ±SE


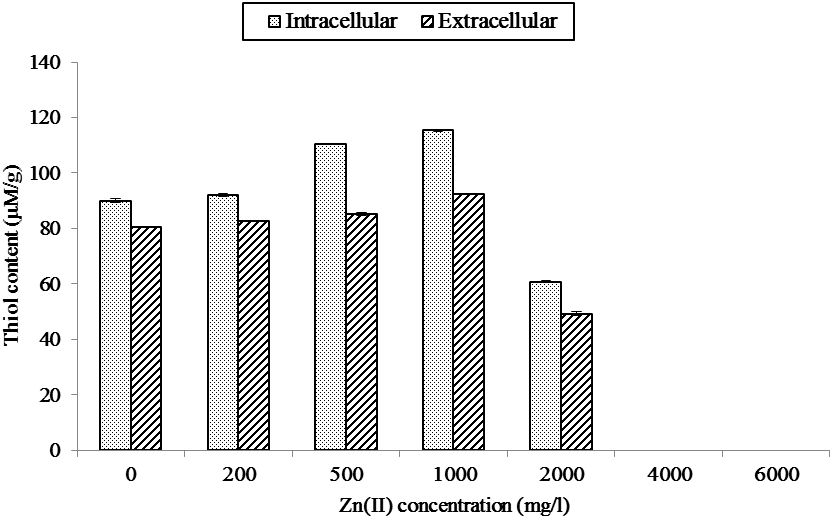


**Fig. 5s:** Extracellular and intracellular thiol contents in *F. solani* under different Zn(II) concentrations. Data represent means of 3 measurements ±SE


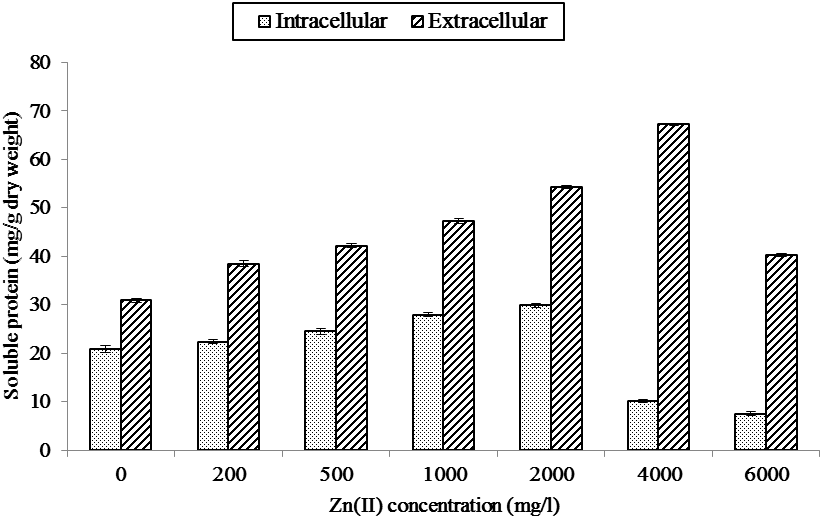


**Fig. 6S**: Extracellular and intracellular protein contents in *F. solani* under different Zn(II) concentrations. Data represent means of 3 measurements ±SE

**
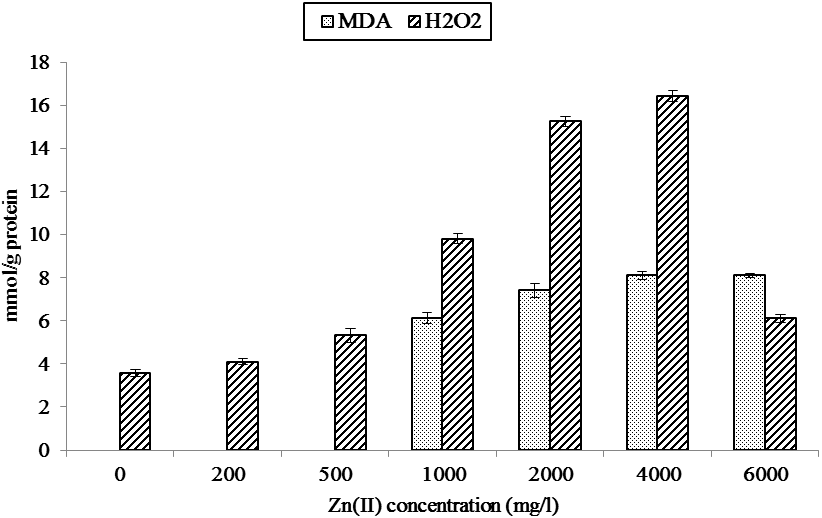
**

**Fig. 7S:** Malonyl dialdehyde (MDA) and Hydrogen peroxide (H_2_O_2_) contents in *F. solani* under different Zn(II) concentrations. Data represent means of 3 measurements ±SE


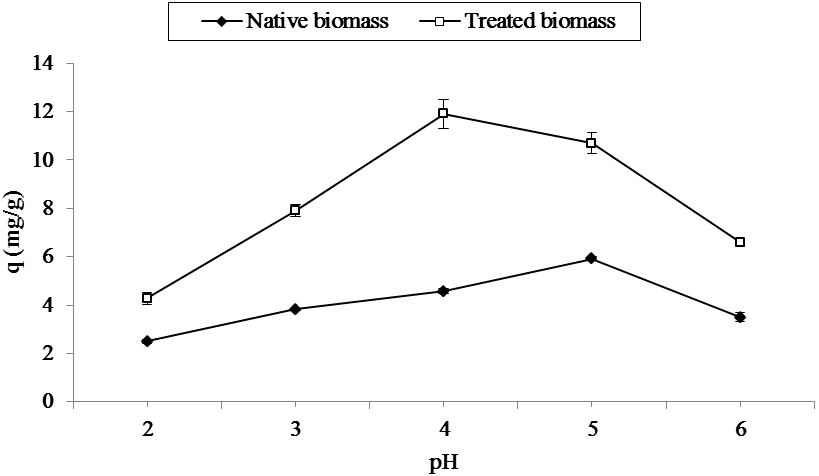


**Fig. 8S**: Effect of initial pH on Zn(II) uptake capacity of native and alkali-treated biomass of *F. solani*. Biosorption conditions: initial Zn(II) concentration =600 mg/l, biosorbent dose=1.0 g/l, contact time 40 min (alkali-treated biomass) and 6 h (native biomass), temperature= 30˚C at 140 rpm


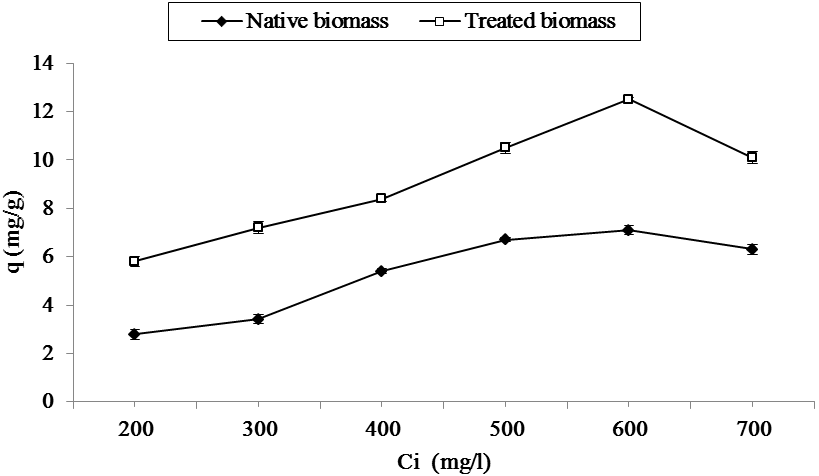


**Fig. 9S:** Effect of initial metal ion concentration (C_i_) on Zn(II) uptake capacity of native and alkali-treated biomass of *F. solani*. Biosorption conditions: initial pH= 4 (alkali-treated biomass) and 5.0 (native biomass), biosorbent dose=1.0 g/l, contact time 40 min (alkali-treated biomass) and 6 h (native biomass), temperature= 30˚C at 140 rpm


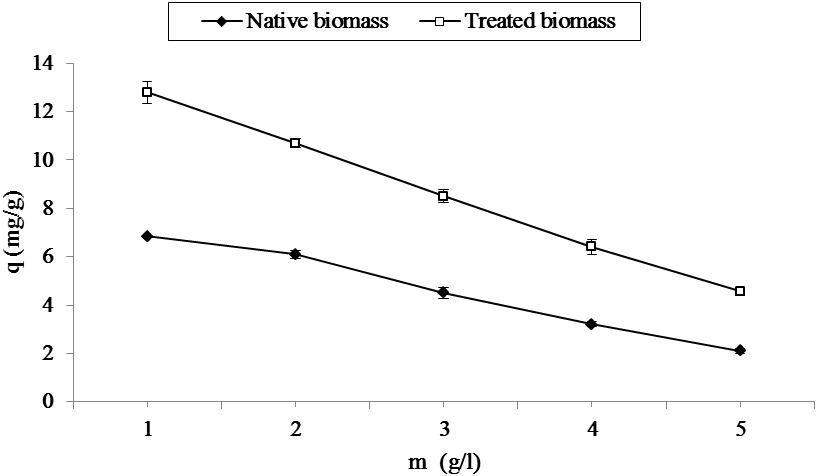


**Fig. 10S:** Effect of initial biosorbent dose (m) (g/l) on Zn(II) uptake capacity of native and alkali-treated biomass of *F. solani*. Biosorption conditions: initial pH= 4 (alkali-treated biomass) and 5.0 (native biomass), contact time 40 min (alkali-treated biomass) and 6 h (native biomass), temperature= 30˚C at 140 rpm


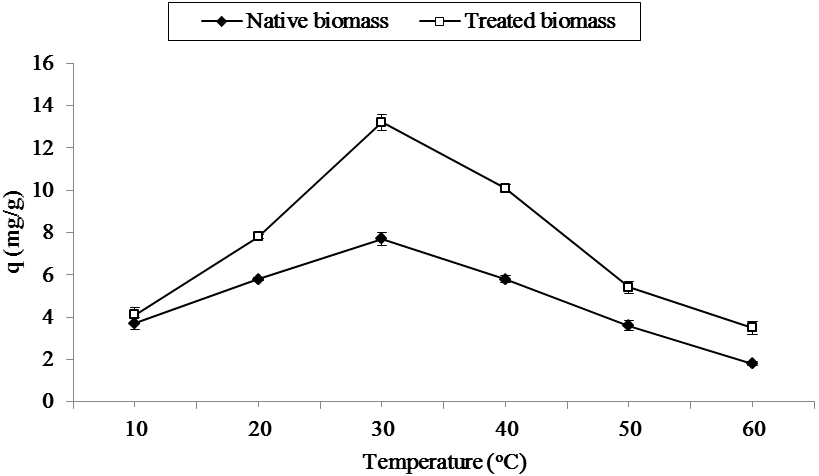


**Fig. 11S**: Effect of temperature (˚C) on Zn(II) uptake capacity of native and alkali-treated biomass of *F. solani*. Biosorption conditions: initial pH= 4 (alkali-treated biomass) and 5.0 (native biomass), contact time 40 min (alkali-treated biomass) and 6 h (native biomass) at 140 rpm


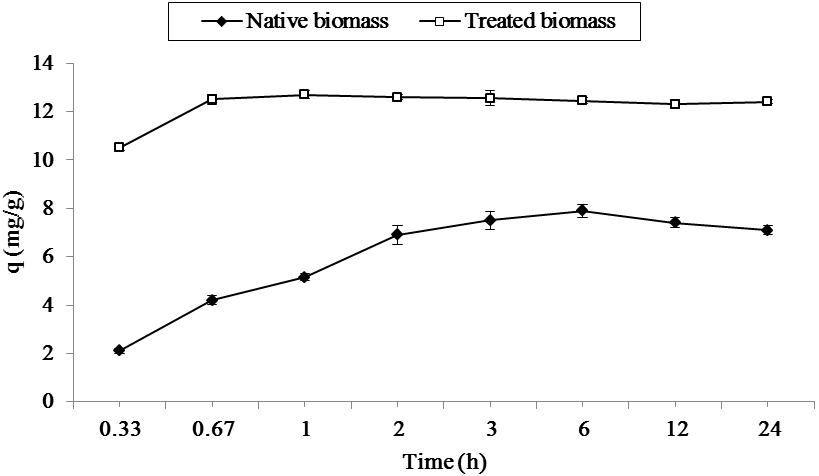


**Fig. 12S:** Effect of contact time (h) on Zn(II) uptake capacity of native and alkali-treated biomass of *F. solani* Biosorption conditions: initial pH= 4 (alkali-treated biomass) and 5.0 (native biomass), initial Zn(II) concentration =600 mg/l, biosorbent dose=1.0 g/l, temperature= 30˚C at 140 rpm
